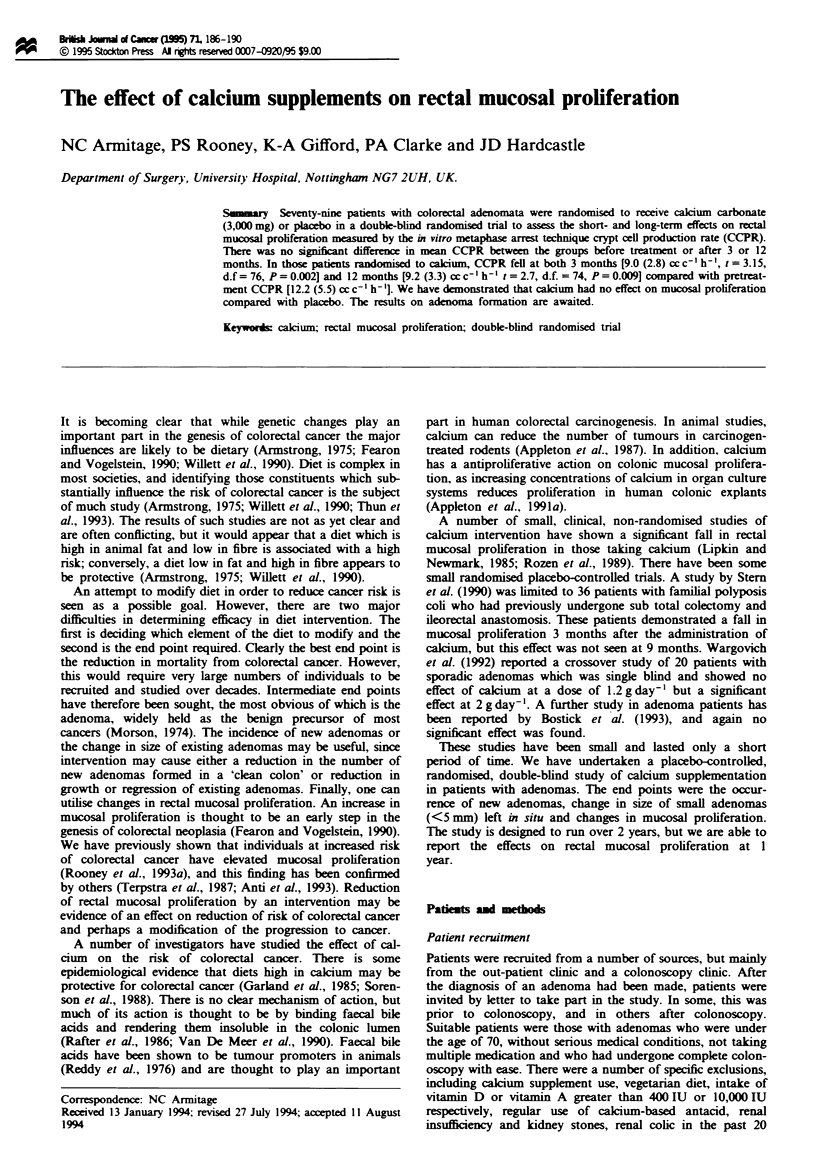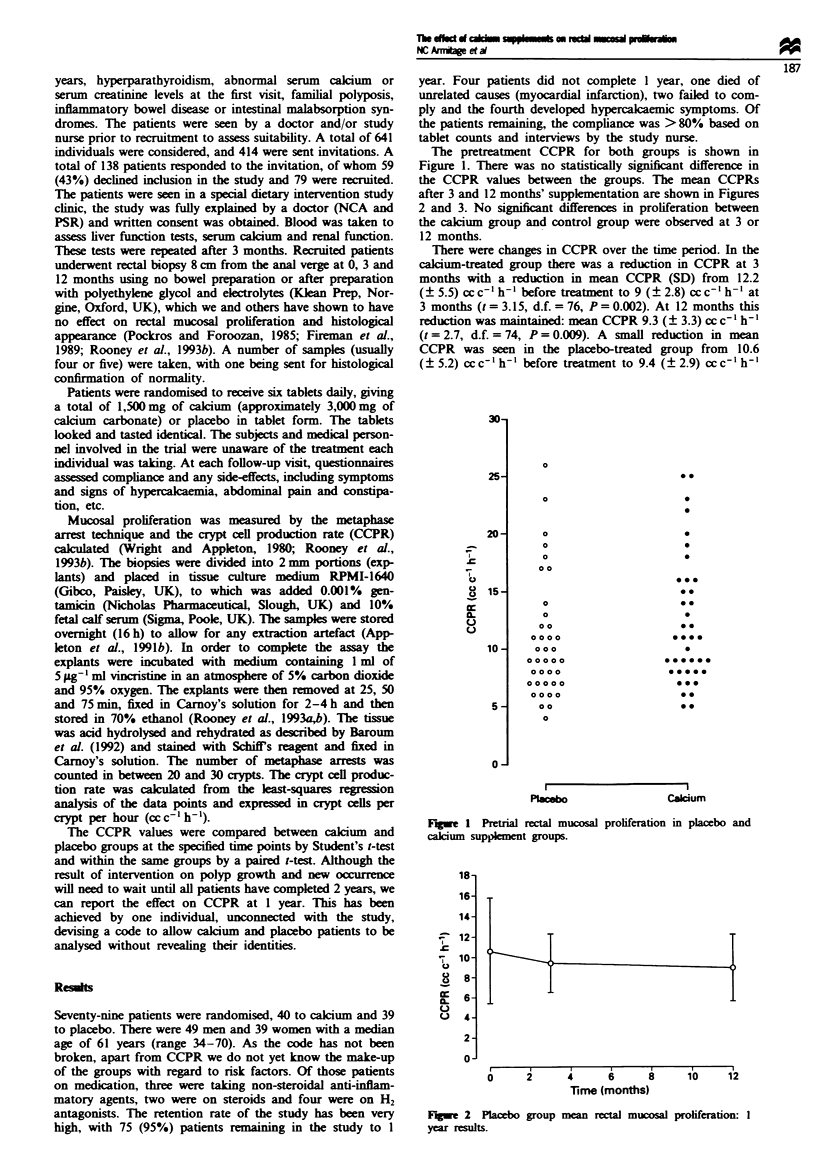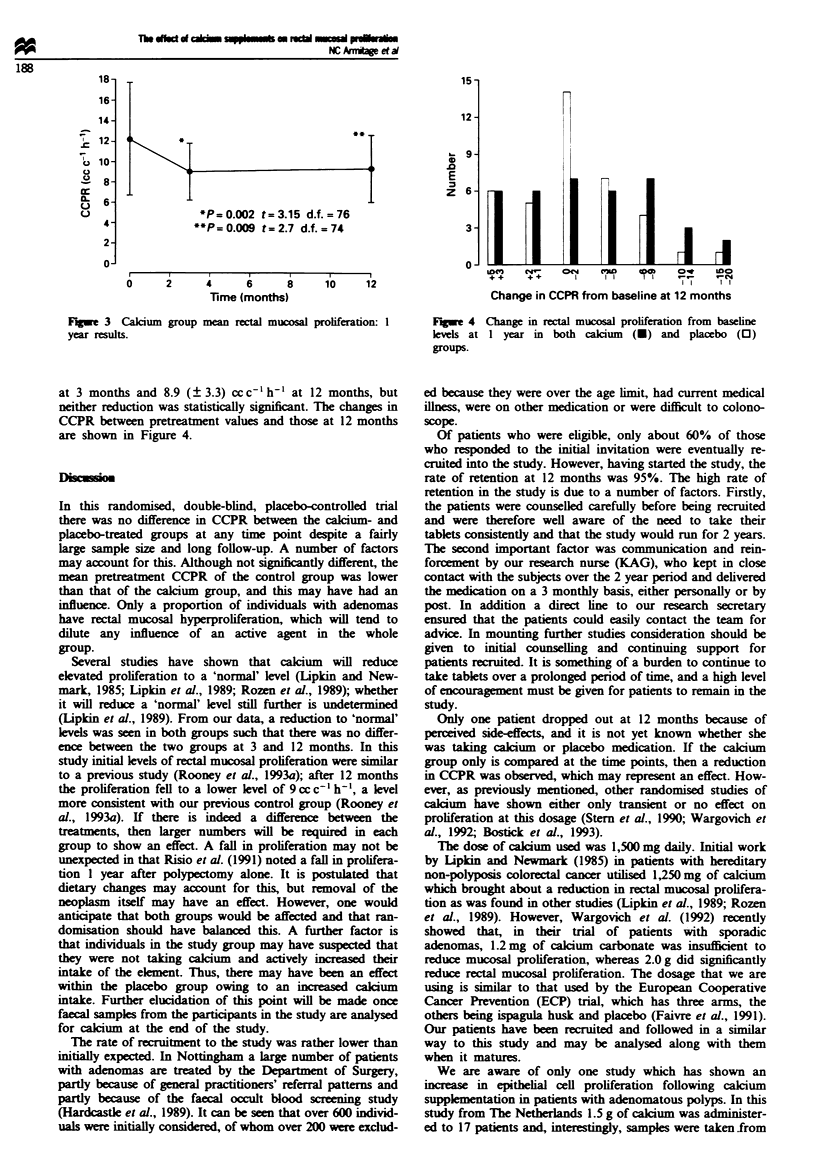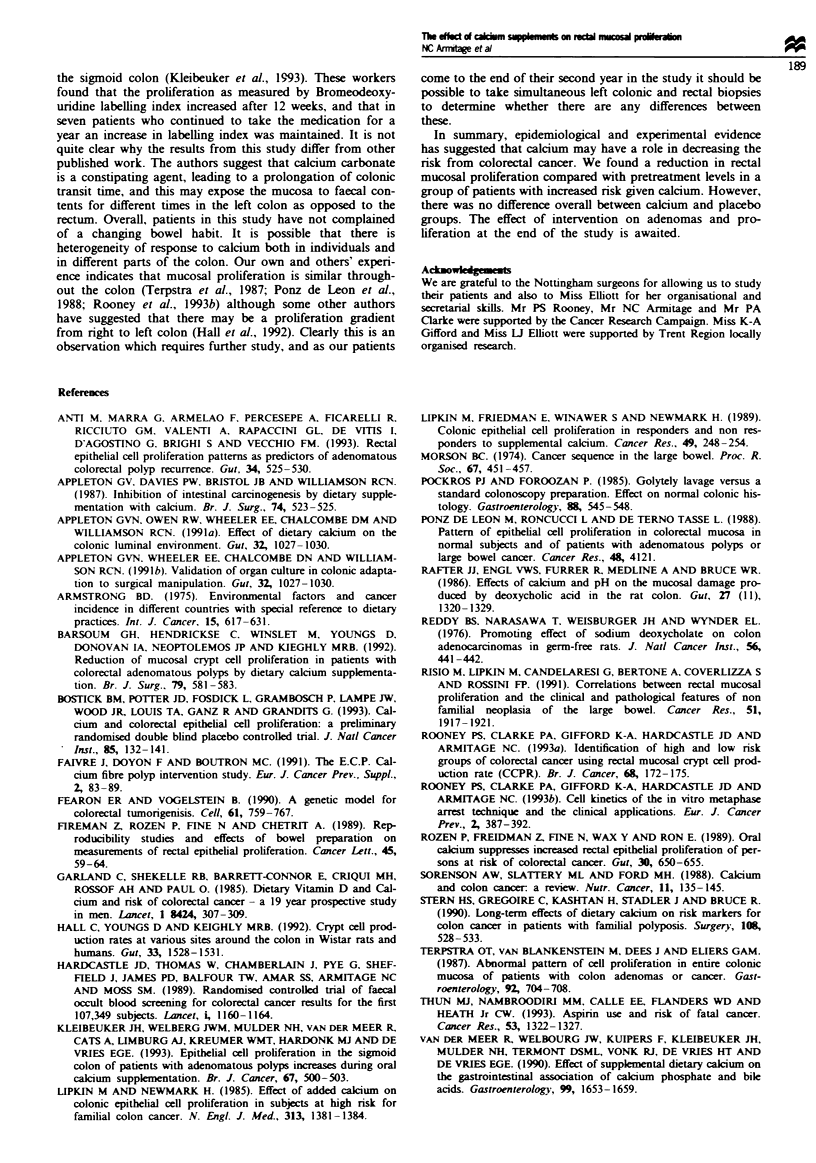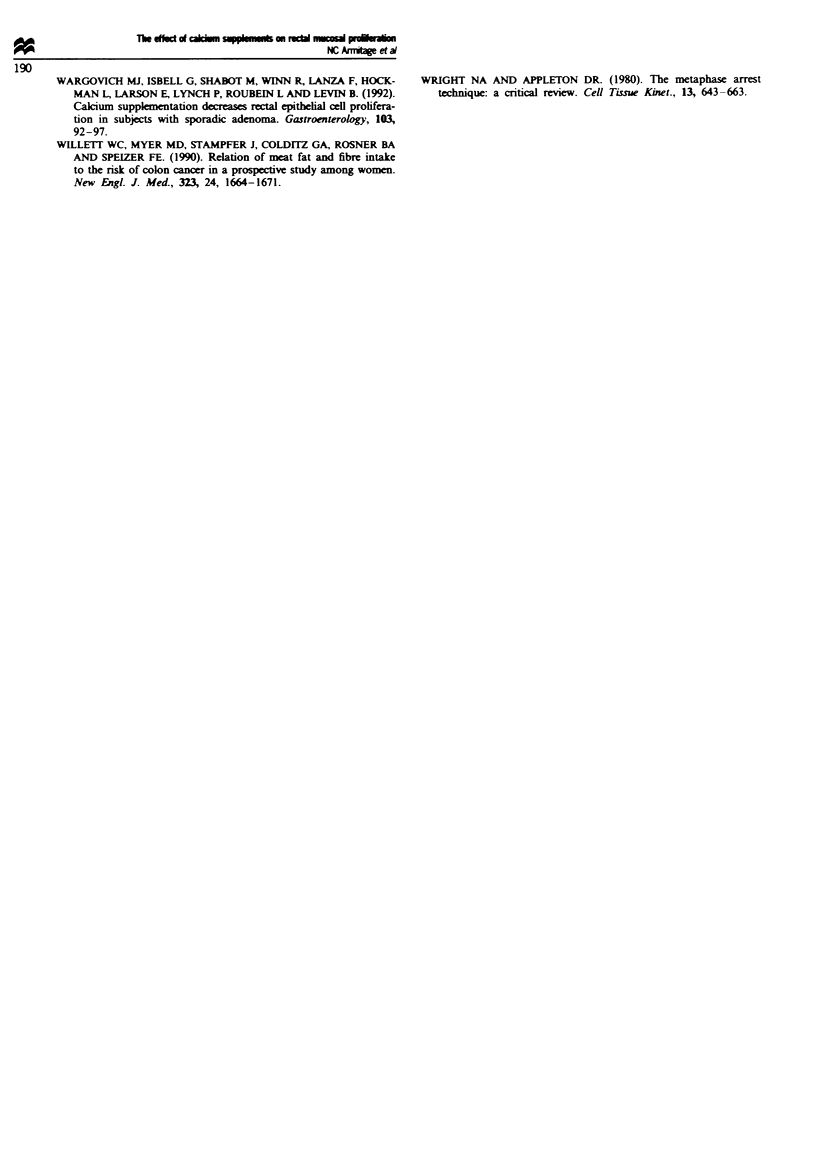# The effect of calcium supplements on rectal mucosal proliferation.

**DOI:** 10.1038/bjc.1995.38

**Published:** 1995-01

**Authors:** N. C. Armitage, P. S. Rooney, K. A. Gifford, P. A. Clarke, J. D. Hardcastle

**Affiliations:** Department of Surgery, University Hospital, Nottingham, UK.

## Abstract

Seventy-nine patients with colorectal adenomata were randomised to receive calcium carbonate (3,000 mg) or placebo in a double-blind randomised trial to assess the short- and long-term effects on rectal mucosal proliferation measured by the in vitro metaphase arrest technique crypt cell production rate (CCPR). There was no significant difference in mean CCPR between the groups before treatment or after 3 or 12 months. In those patients randomised to calcium, CCPR fell at both 3 months [9.0 (2.8) cc c-1 h-1, t = 3.15, d.f = 76, P = 0.002] and 12 months [9.2 (3.3) cc c-1 h-1 t = 2.7, d.f. = 74, P = 0.009] compared with pretreatment CCPR [12.2 (5.5) cc c-1 h-1]. We have demonstrated that calcium had no effect on mucosal proliferation compared with placebo. The results on adenoma formation are awaited.


### The effect of calcium supplements on rectal mucosal proliferation

NC Armitage, PS Rooney, K-A Gifford, PA Clarke and JD Hardcastle

*Department of Surgery, University Hospital, Nottingham NG7 2UH, UK.*

**Summary** Seventy-nine patients with colorectal adenomata were randomised to receive calcium carbonate (3,000 mg) or placebo in a double-blind randomised trial to assess the short- and long-term effects on rectal mucosal proliferation measured by the *in vitro* metaphase arrest technique crypt cell production rate (CCPR). There was no significant difference in mean CCPR between the groups before treatment or after 3 or 12 months. In those patients randomised to calcium, CCPR fell at both 3 months [9.0 (2.8) cc $c^{-1}$ $h^{-1}$, $t = 3.15$, d.f = 76, $P = 0.002$] and 12 months [9.2 (3.3) cc $c^{-1}$ $h^{-1}$ $t = 2.7$, d.f. = 74, $P = 0.009$] compared with pretreatment CCPR [12.2 (5.5) cc $c^{-1}$ $h^{-1}$]. We have demonstrated that calcium had no effect on mucosal proliferation compared with placebo. The results on adenoma formation are awaited.

**Keywords:** calcium; rectal mucosal proliferation; double-blind randomised trial

It is becoming clear that while genetic changes play an important part in the genesis of colorectal cancer the major influences are likely to be dietary (Armstrong, 1975; Fearon and Vogelstein, 1990; Willett *et al.*, 1990). Diet is complex in most societies, and identifying those constituents which substantially influence the risk of colorectal cancer is the subject of much study (Armstrong, 1975; Willett *et al.*, 1990; Thun *et al.*, 1993). The results of such studies are not as yet clear and are often conflicting, but it would appear that a diet which is high in animal fat and low in fibre is associated with a high risk; conversely, a diet low in fat and high in fibre appears to be protective (Armstrong, 1975; Willett *et al.*, 1990).

An attempt to modify diet in order to reduce cancer risk is seen as a possible goal. However, there are two major difficulties in determining efficacy in diet intervention. The first is deciding which element of the diet to modify and the second is the end point required. Clearly the best end point is the reduction in mortality from colorectal cancer. However, this would require very large numbers of individuals to be recruited and studied over decades. Intermediate end points have therefore been sought, the most obvious of which is the adenoma, widely held as the benign precursor of most cancers (Morson, 1974). The incidence of new adenomas or the change in size of existing adenomas may be useful, since intervention may cause either a reduction in the number of new adenomas formed in a 'clean colon' or reduction in growth or regression of existing adenomas. Finally, one can utilise changes in rectal mucosal proliferation. An increase in mucosal proliferation is thought to be an early step in the genesis of colorectal neoplasia (Fearon and Vogelstein, 1990). We have previously shown that individuals at increased risk of colorectal cancer have elevated mucosal proliferation (Rooney *et al.*, 1993*a*), and this finding has been confirmed by others (Terpstra *et al.*, 1987; Anti *et al.*, 1993). Reduction of rectal mucosal proliferation by an intervention may be evidence of an effect on reduction of risk of colorectal cancer and perhaps a modification of the progression to cancer.

A number of investigators have studied the effect of calcium on the risk of colorectal cancer. There is some epidemiological evidence that diets high in calcium may be protective for colorectal cancer (Garland *et al.*, 1985; Sorenson *et al.*, 1988). There is no clear mechanism of action, but much of its action is thought to be by binding faecal bile acids and rendering them insoluble in the colonic lumen (Rafter *et al.*, 1986; Van De Meer *et al.*, 1990). Faecal bile acids have been shown to be tumour promoters in animals (Reddy *et al.*, 1976) and are thought to play an important part in human colorectal carcinogenesis. In animal studies, calcium can reduce the number of tumours in carcinogen-treated rodents (Appleton *et al.*, 1987). In addition, calcium has a antiproliferative action on colonic mucosal proliferation, as increasing concentrations of calcium in organ culture systems reduces proliferation in human colonic explants (Appleton *et al.*, 1991*a*).

A number of small, clinical, non-randomised studies of calcium intervention have shown a significant fall in rectal mucosal proliferation in those taking calcium (Lipkin and Newmark, 1985; Rozen *et al.*, 1989). There have been some small randomised placebo-controlled trials. A study by Stern *et al.* (1990) was limited to 36 patients with familial polyposis coli who had previously undergone sub total colectomy and ileorectal anastomosis. These patients demonstrated a fall in mucosal proliferation 3 months after the administration of calcium, but this effect was not seen at 9 months. Wargovich *et al.* (1992) reported a crossover study of 20 patients with sporadic adenomas which was single blind and showed no effect of calcium at a dose of 1.2 g $day^{-1}$ but a significant effect at 2 g $day^{-1}$. A further study in adenoma patients has been reported by Bostick *et al.* (1993), and again no significant effect was found.

These studies have been small and lasted only a short period of time. We have undertaken a placebo-controlled, randomised, double-blind study of calcium supplementation in patients with adenomas. The end points were the occurrence of new adenomas, change in size of small adenomas (<5 mm) left *in situ* and changes in mucosal proliferation. The study is designed to run over 2 years, but we are able to report the effects on rectal mucosal proliferation at 1 year.

### Patients and methods

### *Patient recruitment*

Patients were recruited from a number of sources, but mainly from the out-patient clinic and a colonoscopy clinic. After the diagnosis of an adenoma had been made, patients were invited by letter to take part in the study. In some, this was prior to colonoscopy, and in others after colonoscopy. Suitable patients were those with adenomas who were under the age of 70, without serious medical conditions, not taking multiple medication and who had undergone complete colonoscopy with ease. There were a number of specific exclusions, including calcium supplement use, vegetarian diet, intake of vitamin D or vitamin A greater than 400 IU or 10,000 IU respectively, regular use of calcium-based antacid, renal insufficiency and kidney stones, renal colic in the past 20

Correspondence: NC Armitage
Received 13 January 1994; revised 27 July 1994; accepted 11 August 1994

years, hyperparathyroidism, abnormal serum calcium or serum creatinine levels at the first visit, familial polyposis, inflammatory bowel disease or intestinal malabsorption syndromes. The patients were seen by a doctor and/or study nurse prior to recruitment to assess suitability. A total of 641 individuals were considered, and 414 were sent invitations. A total of 138 patients responded to the invitation, of whom 59 (43%) declined inclusion in the study and 79 were recruited. The patients were seen in a special dietary intervention study clinic, the study was fully explained by a doctor (NCA and PSR) and written consent was obtained. Blood was taken to assess liver function tests, serum calcium and renal function. These tests were repeated after 3 months. Recruited patients underwent rectal biopsy 8 cm from the anal verge at 0, 3 and 12 months using no bowel preparation or after preparation with polyethylene glycol and electrolytes (Klean Prep, Norgine, Oxford, UK), which we and others have shown to have no effect on rectal mucosal proliferation and histological appearance (Pockros and Foroozan, 1985; Fireman *et al.*, 1989; Rooney *et al.*, 1993*b*). A number of samples (usually four or five) were taken, with one being sent for histological confirmation of normality.

Patients were randomised to receive six tablets daily, giving a total of 1,500 mg of calcium (approximately 3,000 mg of calcium carbonate) or placebo in tablet form. The tablets looked and tasted identical. The subjects and medical personnel involved in the trial were unaware of the treatment each individual was taking. At each follow-up visit, questionnaires assessed compliance and any side-effects, including symptoms and signs of hypercalcaemia, abdominal pain and constipation, etc.

Mucosal proliferation was measured by the metaphase arrest technique and the crypt cell production rate (CCPR) calculated (Wright and Appleton, 1980; Rooney *et al.*, 1993*b*). The biopsies were divided into 2 mm portions (explants) and placed in tissue culture medium RPMI-1640 (Gibco, Paisley, UK), to which was added 0.001% gentamicin (Nicholas Pharmaceutical, Slough, UK) and 10% fetal calf serum (Sigma, Poole, UK). The samples were stored overnight (16 h) to allow for any extraction artefact (Appleton *et al.*, 1991*b*). In order to complete the assay the explants were incubated with medium containing 1 ml of 5 $\mu g^{-1}$ ml vincristine in an atmosphere of 5% carbon dioxide and 95% oxygen. The explants were then removed at 25, 50 and 75 min, fixed in Carnoy's solution for 2–4 h and then stored in 70% ethanol (Rooney *et al.*, 1993*a*,*b*). The tissue was acid hydrolysed and rehydrated as described by Baroum *et al.* (1992) and stained with Schiff's reagent and fixed in Carnoy's solution. The number of metaphase arrests was counted in between 20 and 30 crypts. The crypt cell production rate was calculated from the least-squares regression analysis of the data points and expressed in crypt cells per crypt per hour ($cc\,c^{-1}\,h^{-1}$).

The CCPR values were compared between calcium and placebo groups at the specified time points by Student's *t*-test and within the same groups by a paired *t*-test. Although the result of intervention on polyp growth and new occurrence will need to wait until all patients have completed 2 years, we can report the effect on CCPR at 1 year. This has been achieved by one individual, unconnected with the study, devising a code to allow calcium and placebo patients to be analysed without revealing their identities.

### Results

Seventy-nine patients were randomised, 40 to calcium and 39 to placebo. There were 49 men and 39 women with a median age of 61 years (range 34–70). As the code has not been broken, apart from CCPR we do not yet know the make-up of the groups with regard to risk factors. Of those patients on medication, three were taking non-steroidal anti-inflammatory agents, two were on steroids and four were on $H_2$ antagonists. The retention rate of the study has been very high, with 75 (95%) patients remaining in the study to 1 year. Four patients did not complete 1 year, one died of unrelated causes (myocardial infarction), two failed to comply and the fourth developed hypercalcaemic symptoms. Of the patients remaining, the compliance was >80% based on tablet counts and interviews by the study nurse.

The pretreatment CCPR for both groups is shown in Figure 1. There was no statistically significant difference in the CCPR values between the groups. The mean CCPRs after 3 and 12 months' supplementation are shown in Figures 2 and 3. No significant differences in proliferation between the calcium group and control group were observed at 3 or 12 months.

There were changes in CCPR over the time period. In the calcium-treated group there was a reduction in CCPR at 3 months with a reduction in mean CCPR (SD) from 12.2 (± 5.5) $cc\,c^{-1}\,h^{-1}$ before treatment to 9 (± 2.8) $cc\,c^{-1}\,h^{-1}$ at 3 months ($t = 3.15$, d.f. = 76, $P = 0.002$). At 12 months this reduction was maintained: mean CCPR 9.3 (± 3.3) $cc\,c^{-1}\,h^{-1}$ ($t = 2.7$, d.f. = 74, $P = 0.009$). A small reduction in mean CCPR was seen in the placebo-treated group from 10.6 (± 5.2) $cc\,c^{-1}\,h^{-1}$ before treatment to 9.4 (± 2.9) $cc\,c^{-1}\,h^{-1}$

**Figure 1** Pretrial rectal mucosal proliferation in placebo and calcium supplement groups.

**Figure 2** Placebo group mean rectal mucosal proliferation: 1 year results.

**Figure 3** Calcium group mean rectal mucosal proliferation: 1 year results.

**Figure 4** Change in rectal mucosal proliferation from baseline levels at 1 year in both calcium (■) and placebo (□) groups.

at 3 months and 8.9 (± 3.3) $cc\,c^{-1}\,h^{-1}$ at 12 months, but neither reduction was statistically significant. The changes in CCPR between pretreatment values and those at 12 months are shown in Figure 4.

### Discussion

In this randomised, double-blind, placebo-controlled trial there was no difference in CCPR between the calcium- and placebo-treated groups at any time point despite a fairly large sample size and long follow-up. A number of factors may account for this. Although not significantly different, the mean pretreatment CCPR of the control group was lower than that of the calcium group, and this may have had an influence. Only a proportion of individuals with adenomas have rectal mucosal hyperproliferation, which will tend to dilute any influence of an active agent in the whole group.

Several studies have shown that calcium will reduce elevated proliferation to a 'normal' level (Lipkin and Newmark, 1985; Lipkin *et al.*, 1989; Rozen *et al.*, 1989); whether it will reduce a 'normal' level still further is undetermined (Lipkin *et al.*, 1989). From our data, a reduction to 'normal' levels was seen in both groups such that there was no difference between the two groups at 3 and 12 months. In this study initial levels of rectal mucosal proliferation were similar to a previous study (Rooney *et al.*, 1993*a*); after 12 months the proliferation fell to a lower level of 9 $cc\,c^{-1}\,h^{-1}$, a level more consistent with our previous control group (Rooney *et al.*, 1993*a*). If there is indeed a difference between the treatments, then larger numbers will be required in each group to show an effect. A fall in proliferation may not be unexpected in that Risio *et al.* (1991) noted a fall in proliferation 1 year after polypectomy alone. It is postulated that dietary changes may account for this, but removal of the neoplasm itself may have an effect. However, one would anticipate that both groups would be affected and that randomisation should have balanced this. A further factor is that individuals in the study group may have suspected that they were not taking calcium and actively increased their intake of the element. Thus, there may have been an effect within the placebo group owing to an increased calcium intake. Further elucidation of this point will be made once faecal samples from the participants in the study are analysed for calcium at the end of the study.

The rate of recruitment to the study was rather lower than initially expected. In Nottingham a large number of patients with adenomas are treated by the Department of Surgery, partly because of general practitioners' referral patterns and partly because of the faecal occult blood screening study (Hardcastle *et al.*, 1989). It can be seen that over 600 individuals were initially considered, of whom over 200 were excluded because they were over the age limit, had current medical illness, were on other medication or were difficult to colonoscope.

Of patients who were eligible, only about 60% of those who responded to the initial invitation were eventually recruited into the study. However, having started the study, the rate of retention at 12 months was 95%. The high rate of retention in the study is due to a number of factors. Firstly, the patients were counselled carefully before being recruited and were therefore well aware of the need to take their tablets consistently and that the study would run for 2 years. The second important factor was communication and reinforcement by our research nurse (KAG), who kept in close contact with the subjects over the 2 year period and delivered the medication on a 3 monthly basis, either personally or by post. In addition a direct line to our research secretary ensured that the patients could easily contact the team for advice. In mounting further studies consideration should be given to initial counselling and continuing support for patients recruited. It is something of a burden to continue to take tablets over a prolonged period of time, and a high level of encouragement must be given for patients to remain in the study.

Only one patient dropped out at 12 months because of perceived side-effects, and it is not yet known whether she was taking calcium or placebo medication. If the calcium group only is compared at the time points, then a reduction in CCPR was observed, which may represent an effect. However, as previously mentioned, other randomised studies of calcium have shown either only transient or no effect on proliferation at this dosage (Stern *et al.*, 1990; Wargovich *et al.*, 1992; Bostick *et al.*, 1993).

The dose of calcium used was 1,500 mg daily. Initial work by Lipkin and Newmark (1985) in patients with hereditary non-polyposis colorectal cancer utilised 1,250 mg of calcium which brought about a reduction in rectal mucosal proliferation as was found in other studies (Lipkin *et al.*, 1989; Rozen *et al.*, 1989). However, Wargovich *et al.* (1992) recently showed that, in their trial of patients with sporadic adenomas, 1.2 mg of calcium carbonate was insufficient to reduce mucosal proliferation, whereas 2.0 g did significantly reduce rectal mucosal proliferation. The dosage that we are using is similar to that used by the European Cooperative Cancer Prevention (ECP) trial, which has three arms, the others being ispagula husk and placebo (Faivre *et al.*, 1991). Our patients have been recruited and followed in a similar way to this study and may be analysed along with them when it matures.

We are aware of only one study which has shown an increase in epithelial cell proliferation following calcium supplementation in patients with adenomatous polyps. In this study from The Netherlands 1.5 g of calcium was administered to 17 patients and, interestingly, samples were taken from the sigmoid colon (Kleibeuker *et al.*, 1993). These workers found that the proliferation as measured by Bromeodeoxyuridine labelling index increased after 12 weeks, and that in seven patients who continued to take the medication for a year an increase in labelling index was maintained. It is not quite clear why the results from this study differ from other published work. The authors suggest that calcium carbonate is a constipating agent, leading to a prolongation of colonic transit time, and this may expose the mucosa to faecal contents for different times in the left colon as opposed to the rectum. Overall, patients in this study have not complained of a changing bowel habit. It is possible that there is heterogeneity of response to calcium both in individuals and in different parts of the colon. Our own and others' experience indicates that mucosal proliferation is similar throughout the colon (Terpstra *et al.*, 1987; Ponz de Leon *et al.*, 1988; Rooney *et al.*, 1993*b*) although some other authors have suggested that there may be a proliferation gradient from right to left colon (Hall *et al.*, 1992). Clearly this is an observation which requires further study, and as our patients come to the end of their second year in the study it should be possible to take simultaneous left colonic and rectal biopsies to determine whether there are any differences between these.

In summary, epidemiological and experimental evidence has suggested that calcium may have a role in decreasing the risk from colorectal cancer. We found a reduction in rectal mucosal proliferation compared with pretreatment levels in a group of patients with increased risk given calcium. However, there was no difference overall between calcium and placebo groups. The effect of intervention on adenomas and proliferation at the end of the study is awaited.

**Acknowledgements**
We are grateful to the Nottingham surgeons for allowing us to study their patients and also to Miss Elliott for her organisational and secretarial skills. Mr PS Rooney, Mr NC Armitage and Mr PA Clarke were supported by the Cancer Research Campaign. Miss K-A Gifford and Miss LJ Elliott were supported by Trent Region locally organised research.

**References**

ANTI M, MARRA G, ARMELAO F, PERCESEPE A, FICARELLI R, RICCIUTO GM, VALENTI A, RAPACCINI GL, DE VITIS I, D'AGOSTINO G, BRIGHI S AND VECCHIO FM. (1993). Rectal epithelial cell proliferation patterns as predictors of adenomatous colorectal polyp recurrence. *Gut*, **34**, 525-530.

APPLETON GV, DAVIES PW, BRISTOL JB AND WILLIAMSON RCN. (1987). Inhibition of intestinal carcinogenesis by dietary supplementation with calcium. *Br. J. Surg.*, **74**, 523-525.

APPLETON GVN, OWEN RW, WHEELER EE, CHALCOMBE DM AND WILLIAMSON RCN. (1991*a*). Effect of dietary calcium on the colonic luminal environment. *Gut*, **32**, 1027-1030.

APPLETON GVN, WHEELER EE, CHALCOMBE DN AND WILLIAMSON RCN. (1991*b*). Validation of organ culture in colonic adaptation to surgical manipulation. *Gut*, **32**, 1027-1030.

ARMSTRONG BD. (1975). Environmental factors and cancer incidence in different countries with special reference to dietary practices. *Int. J. Cancer*, **15**, 617-631.

BARSOUM GH, HENDRICKSE C, WINSLET M, YOUNGS D, DONOVAN IA, NEOPTOLEMOS JP AND KIEGHLY MRB. (1992). Reduction of mucosal crypt cell proliferation in patients with colorectal adenomatous polyps by dietary calcium supplementation. *Br. J. Surg.*, **79**, 581-583.

BOSTICK BM, POTTER JD, FOSDICK L, GRAMBOSCH P, LAMPE JW, WOOD JR, LOUIS TA, GANZ R AND GRANDITS G. (1993). Calcium and colorectal epithelial cell proliferation: a preliminary randomised double blind placebo controlled trial. *J. Natl Cancer Inst.*, **85**, 132-141.

FAIVRE J, DOYON F AND BOUTRON MC. (1991). The E.C.P. Calcium fibre polyp intervention study. *Eur. J. Cancer Prev., Suppl.*, **2**, 83-89.

FEARON ER AND VOGELSTEIN B. (1990). A genetic model for colorectal tumorigenisis. *Cell*, **61**, 759-767.

FIREMAN Z, ROZEN P, FINE N AND CHETRIT A. (1989). Reproducibility studies and effects of bowel preparation on measurements of rectal epithelial proliferation. *Cancer Lett.*, **45**, 59-64.

GARLAND C, SHEKELLE RB, BARRETT-CONNOR E, CRIQUI MH, ROSSOF AH AND PAUL O. (1985). Dietary Vitamin D and Calcium and risk of colorectal cancer - a 19 year prospective study in men. *Lancet*, **1 8424**, 307-309.

HALL C, YOUNGS D AND KEIGHLY MRB. (1992). Crypt cell production rates at various sites around the colon in Wistar rats and humans. *Gut*, **33**, 1528-1531.

HARDCASTLE JD, THOMAS W, CHAMBERLAIN J, PYE G, SHEFFIELD J, JAMES PD, BALFOUR TW, AMAR SS, ARMITAGE NC AND MOSS SM. (1989). Randomised controlled trial of faecal occult blood screening for colorectal cancer results for the first 107,349 subjects. *Lancet*, **i**, 1160-1164.

KLEIBEUKER JH, WELBERG JWM, MULDER NH, VAN DER MEER R, CATS A, LIMBURG AJ, KREUMER WMT, HARDONK MJ AND DE VRIES EGE. (1993). Epithelial cell proliferation in the sigmoid colon of patients with adenomatous polyps increases during oral calcium supplementation. *Br. J. Cancer*, **67**, 500-503.

LIPKIN M AND NEWMARK H. (1985). Effect of added calcium on colonic epithelial cell proliferation in subjects at high risk for familial colon cancer. *N. Engl. J. Med.*, **313**, 1381-1384.

LIPKIN M, FRIEDMAN E, WINAWER S AND NEWMARK H. (1989). Colonic epithelial cell proliferation in responders and non responders to supplemental calcium. *Cancer Res.*, **49**, 248-254.

MORSON BC. (1974). Cancer sequence in the large bowel. *Proc. R. Soc.*, **67**, 451-457.

POCKROS PJ AND FOROOZAN P. (1985). Golytely lavage versus a standard colonoscopy preparation. Effect on normal colonic histology. *Gastroenterology*, **88**, 545-548.

PONZ DE LEON M, RONCUCCI L AND DE TERNO TASSE L. (1988). Pattern of epithelial cell proliferation in colorectal mucosa in normal subjects and of patients with adenomatous polyps or large bowel cancer. *Cancer Res.*, **48**, 4121.

RAFTER JJ, ENGL VWS, FURRER R, MEDLINE A AND BRUCE WR. (1986). Effects of calcium and pH on the mucosal damage produced by deoxycholic acid in the rat colon. *Gut*, **27** (11), 1320-1329.

REDDY BS, NARASAWA T, WEISBURGER JH AND WYNDER EL. (1976). Promoting effect of sodium deoxycholate on colon adenocarcinomas in germ-free rats. *J. Natl Cancer Inst.*, **56**, 441-442.

RISIO M, LIPKIN M, CANDELARESI G, BERTONE A, COVERLIZZA S AND ROSSINI FP. (1991). Correlations between rectal mucosal proliferation and the clinical and pathological features of non familial neoplasia of the large bowel. *Cancer Res.*, **51**, 1917-1921.

ROONEY PS, CLARKE PA, GIFFORD K-A, HARDCASTLE JD AND ARMITAGE NC. (1993*a*). Identification of high and low risk groups of colorectal cancer using rectal mucosal crypt cell production rate (CCPR). *Br. J. Cancer*, **68**, 172-175.

ROONEY PS, CLARKE PA, GIFFORD K-A, HARDCASTLE JD AND ARMITAGE NC. (1993*b*). Cell kinetics of the in vitro metaphase arrest technique and the clinical applications. *Eur. J. Cancer Prev.*, **2**, 387-392.

ROZEN P, FREIDMAN Z, FINE N, WAX Y AND RON E. (1989). Oral calcium suppresses increased rectal epithelial proliferation of persons at risk of colorectal cancer. *Gut*, **30**, 650-655.

SORENSON AW, SLATTERY ML AND FORD MH. (1988). Calcium and colon cancer: a review. *Nutr. Cancer*, **11**, 135-145.

STERN HS, GREGOIRE C, KASHTAN H, STADLER J AND BRUCE R. (1990). Long-term effects of dietary calcium on risk markers for colon cancer in patients with familial polyposis. *Surgery*, **108**, 528-533.

TERPSTRA OT, VAN BLANKENSTEIN M, DEES J AND ELIERS GAM. (1987). Abnormal pattern of cell proliferation in entire colonic mucosa of patients with colon adenomas or cancer. *Gastroenterology*, **92**, 704-708.

THUN MJ, NAMBROODIRI MM, CALLE EE, FLANDERS WD AND HEATH Jr CW. (1993). Aspirin use and risk of fatal cancer. *Cancer Res.*, **53**, 1322-1327.

VAN DER MEER R, WELBOURG JW, KUIPERS F, KLEIBEUKER JH, MULDER NH, TERMONT DSML, VONK RJ, DE VRIES HT AND DE VRIES EGE. (1990). Effect of supplemental dietary calcium on the gastrointestinal association of calcium phosphate and bile acids. *Gastroenterology*, **99**, 1653-1659.

WARGOVICH MJ, ISBELL G, SHABOT M, WINN R, LANZA F, HOCKMAN L, LARSON E, LYNCH P, ROUBEIN L AND LEVIN B. (1992). Calcium supplementation decreases rectal epithelial cell proliferation in subjects with sporadic adenoma. *Gastroenterology*, **103**, 92–97.

WILLETT WC, MYER MD, STAMPFER J, COLDITZ GA, ROSNER BA AND SPEIZER FE. (1990). Relation of meat fat and fibre intake to the risk of colon cancer in a prospective study among women. *New Engl. J. Med.*, **323**, 24, 1664–1671.

WRIGHT NA AND APPLETON DR. (1980). The metaphase arrest technique: a critical review. *Cell Tissue Kinet.*, **13**, 643–663.